# MaxEnt Modeling of the Impacts of Human Activities and Climate Change on the Potential Distribution of *Plantago* in China

**DOI:** 10.3390/biology14050564

**Published:** 2025-05-17

**Authors:** Da Liao, Bing Zhou, Haiyan Xiao, Yuxin Zhang, Shujian Zhang, Qitao Su, Xiaohong Yan

**Affiliations:** Key Laboratory of Jiangxi Province for Biological Invasion and Biosecurity, School of Life Sciences, Jinggangshan University, Ji’an 343009, China; 2308301039@jgsu.edu.cn (D.L.); zhoubing@jgsu.edu.cn (B.Z.); 2308301036@jgsu.edu.cn (H.X.); 2308301009@jgsu.edu.cn (Y.Z.); 2308301045@jgsu.edu.cn (S.Z.)

**Keywords:** biodiversity conservation, climate scenarios, MaxEnt, potential distribution

## Abstract

Human activities exert both beneficial and detrimental impacts on the ecosystem. In recent years, greenhouse gas emissions have significantly increased due to global climate change, causing profound alterations in ecosystem distribution and productivity. The synergistic interplay between climatic shifts and anthropogenic activities is intensifying ecological transformations and disturbances, and accelerating biodiversity depletion. The *Plantago* genus (*Plantaginaceae* family) includes 14 herbaceous species among China’s flora. This study was conducted to elucidate the spatial distribution of *Plantago* species patterns across China and evaluate their differential responses to impending climate change and human interventions. In this study, we projected the potential distributions of *Plantago* species under three climate scenarios (SSP126, SSP245, and SSP585) across current and future temporal intervals (2021–2040, 2041–2060, 2061–2080, and 2081–2100) using the MaxEnt model integrated with ArcGIS V10.8 spatial analysis. A spatial trend analyses was also conducted to assess habitat suitability dynamics by incorporating anthropogenic influence parameters. The model validation yielded AUC values exceeding 0.9, demonstrating excellent model performance and predictive reliability. Precipitation variability and anthropogenic pressure emerged as the most predominant determinants shaping *Plantago* distributions. Centroid migration analyses further indicated the progressive northward displacement of optimal habitats under the projected climate scenarios. These findings significantly advance our understanding of *Plantago* species’ adaptive responses to environmental changes. This study also offers an invaluable scientific foundation for sustainable resource management and ecological conservation strategies.

## Simple Summary

Human activities and climate change threaten ecosystems, risking biodiversity loss and disrupting plant survival. This study examines how 14 medicinal *Plantago* species in China may respond to future climate shifts and human pressures. Using the MaxEnt model and geographic mapping, we predicted where these plants could grow under different climate change scenarios from 2021 to 2100. Our reliable models showed that rainfall changes and human activities most strongly affect their distribution habitats. Worryingly, many species’ suitable habitats will shrink and shift northward as temperatures rise, with these habitats potentially disappearing from their current regions. This highlights climate change’s growing pressure on plant survival. By identifying vulnerable species and high-risk areas, our findings guide targeted conservation efforts to protect these ecologically and medicinally important plants. Farmers, policymakers, and conservationists can use these predictions to prioritize habitat protection, plan sustainable harvesting, and adapt land use practices. This research ultimately supports biodiversity conservation and ensures these plants will remain available for use by future generations in medicine, agriculture, and healthy ecosystems.

## 1. Introduction

China is rich in unique biodiversity resources and represents a strategic focal point for international biodiversity conservation initiatives and advanced ecological research [1]. However, ongoing climate change is seriously threatening this biodiversity. The shifts in future temperature and precipitation will not only adversely impact ecosystems but also cause changes in species distribution ranges [2]. These biogeographical transformations are projected to exert substantial impacts on biodiversity patterns at regional scales. For instance, alterations in precipitation regimes have already modulated the potential distribution of Manchurian maple (*Acer tegmentosum*) and Korean maple (*Acer pseudosieboldianum*) [3]. Anthropogenic perturbations also exert profound impacts on floristic diversity dynamics [4]. These anthropogenic pressures encompass habitat degradation, landscape fragmentation, resource overexploitation, and chemical contamination, which together poses threats to species diversity, thereby impairing essential ecosystem functions [5].

Thus, the rigorous assessment of future climate change impacts on phylogeographic patterns—with particular emphasis on the *Plantago* genus—is imperative to delineate high-risk zones and project biogeographical shifts. The conservation of botanical resources, implementation of sustainable utilization protocols, and pre-emptive mitigation of biological invasions constitute critical imperatives for preserving biodiversity and safeguarding ecosystem service provisioning.

Species distribution modeling (SDM) is fundamentally grounded in ecological niche theory and is also known as ecological niche modeling (ENM) or species habitat modeling (SHM). This variation in nomenclature reflects researchers’ conceptual emphasis within specific types of ecological investigations [6]. The fundamental principle underlying these models revolves around employing computational algorithms to establish statistical relationships between species occurrence records and associated environmental predictors. These models enable spatial extrapolation through geospatial analysis to both reconstruct current distribution patterns and simulate potential habitat suitability under alternative environmental scenarios. Critical model inputs include verified species presence data and ecologically meaningful environmental covariates, which constitute significant components of the multivariate predictive framework characterizing species–environment interactions. This analytical approach facilitates a comprehensive understanding of niche parameters while generating spatially explicit projections applicable to conservation planning and climate change impact assessments [7].

The MaxEnt model is a statistical model known as the maximum entropy model. This model is based on the principle of maximum entropy and uses relevant environmental variables and the known distribution data of a species to project the potential distribution area of that species. The model then evaluates the accuracy of the predicted results by comparing them with actual observations [8]. The maximum entropy (MaxEnt) modeling framework is a probabilistic model that operates by deriving probability distributions. These distributions are designed to maximize information entropy but are constrained by the available empirical data. This approach ensures that the model predictions offer maximal predictive uncertainty. Crucially, this model generates spatially explicit uncertainty quantifications alongside probabilistic habitat suitability projections. Comparative analyses across taxonomic groups consistently validate this model’s superior predictive performance, as measured by AUC (Area Under the Curve) validation metrics [9]. The Maximum Entropy (MaxEnt) modeling framework was implemented in a representative application examining Chongqing’s metropolitan core. This algorithm quantifies the importance of environmental variables through a permutation-based feature contribution analysis coupled with a systematic conservation gap assessment. This integrated methodology enables the simultaneous determination of the influence hierarchies for ecological factors and a spatial prioritization evaluation of existing nature reserves. This model also employs landscape connectivity metrics and habitat suitability thresholds to objectively measure the protected area network’s adequacy within the urban ecological matrix [10]. Recent research initiatives have implemented species distribution modeling to project climatically suitable habitats for *Ligusticum chuanxiong* across China. These authors identified key bioclimatic determinants governing this species’ spatial patterns under both baseline conditions and multiple climate change projections. These studies employed ensemble modeling techniques to quantify the relative influence of critical environmental variables and also simulated potential distributional shifts through time-sliced climate scenarios, thereby elucidating the species’ ecological adaptability within dynamic atmospheric regimes [11].

The *Plantago* genus (*Plantaginaceae* family) contains perennial or annual herbaceous species that predominantly inhabit moist environments. This genus is also distinguished by its linear to lanceolate foliage, spicate inflorescences, minute seeds, and recognized medicinal value. These plants demonstrate broad soil tolerance and frequently colonize grasslands, riparian zones, wetlands, drainage ditches, and other hygric habitats [12]. This genus includes over 190 species distributed globally across temperate to tropical biomes, extending to northward to Arctic latitudes. This genus includes 14 documented species in China, three of which are *Plantago virginica* L. [13], *Plantago aristate* L. [14], and *Plantago lanceolata* L. [15]. These are invasive plant species (IPS) defined as non-native flora capable of autonomous reproduction and dispersal in novel environments. These species also exert detrimental impacts on indigenous ecosystems, economic systems, and social infrastructures. IPS frequently displace native vegetation through habitat monopolization, ultimately precipitating biodiversity erosion and ecological destabilization [16]. Additionally, certain *Plantago* species hold medicinal value, underscoring their socioeconomic importance. Despite these roles, previous research has disproportionately focused on phytochemical and pharmacological factors, leaving critical gaps in the understanding of the spatial dynamics of these species under climate change and anthropogenic pressures [17]. In this study, we employed species distribution modeling through MaxEnt integrated with ArcGIS V10.8 spatial analysis. This investigation specifically analyzed the following: (1) the biogeographic range dynamics of *Plantago* under baseline versus climate-altered conditions; (2) how the spatial patterns of species diversity and endemism in *Plantago* have been reconfigured under the combined effects of climate change and human activity pressures across their distribution ranges; (3) centroid migration trajectories under compounded environmental stressors; and (4) the environmental drivers modulating habitat suitability gradients. To address these objectives, we argue that precipitation and human activities are the primary drivers of *Plantago* distribution shifts under future climate scenarios and put forward the following hypotheses for testing: (1) climate warming will cause the habitats of *Plantago* to shift toward the poles, with invasive species spreading more rapidly than native ones; (2) precipitation extremes and human activities will have a more significant impact on distributional limits; (3) future niche overlaps will intensify the competition between invasive and native taxa; and (4) centroid migration rates will mirror species-specific adaptive capacities.

## 2. Materials and Methods

### 2.1. Acquisition and Processing of Plantago

Distribution data were obtained from the following sources: Global Biodiversity Information Facility, GBIF (https://www.gbif.org, accessed on 21 March 2024); the Plant Photo Bank of China, PPBC (http://ppbc.iplant.cn/, accessed on 21 March 2024); Chinese Field Herbarium (http://www.cfh.ac.cn/, accessed on 21 March 2024), CFH; and National Plant Specimen Resource Center, NPSRC (https://www.cvh.ac.cn/, accessed on 21 March 2024), totaling 9654 records. After removing duplicate points, 6017 unique records were retained. To enhance data accuracy and mitigate overfitting risks, Excel-based data filtering was systematically applied to eliminate questionable records from the dataset. This process specifically targeted duplicate entries from disparate data sources and removed observations with overlapping spatial distributions that lacked precise geocoordinates.

### 2.2. Environmental Data Sources

Climate data were obtained from the World Climate Database (https://www.worldclim.org/, accessed on 21 March 2024). Environmental factors for current and future climate scenarios were obtained at a spatial resolution of 2.5 arcminute (approximately 5 km^2^) with CMIP6, covering three different representative concentration pathways. These pathways were quantified for shared socioeconomic pathway (SSP) scenarios (SSP126, SSP245, and SSP585) covering the time periods of the 2030s (2021–2040), 2050s (2041–2060), 2070s (2061–2080), and 2090s (2081–2100). SSP126 is a sustainable development pathway that emphasizes global cooperation and the use of renewable energy, projecting global warming of about 1.8 °C. SSP245 reflects current policy trends, with emissions stabilizing by the mid-century and an estimated warming of approximately 2.7 °C. In contrast, SSP585 is a high-emission scenario marked by dependence on fossil fuels and limited climate action, which could lead to a temperature rise of around 4.4 °C by 2100. These data are based on the CCSM4 Global Climate Model (GCM) and include both current and projected climate scenarios for the period of the 2090s (2081–2100) [17].

### 2.3. Preprocessing of Environmental Factors

The 23 environmental factors were modeled using the MaxEnt model [18] (Table 1), while topographic variables (elevation, slope, and aspect) were derived from the SRTM 30 m DEM [19]. Human activity (ha) was sourced from SEDAC’s Global Human Footprint dataset (1 km^2^ resolution), integrating population density, land use intensity, and infrastructure metrics [20]. (SEDAC: (http://sedac.ciesin.columbia.edu/wildareas/) accessed on 21 March 2024). The SEDAC database provides spatially explicit global human footprint data, integrating multiple human activity pressure layers to quantify cumulative human impacts on terrestrial ecosystems.

### 2.4. Model Accuracy Test

A vector map of China from the website of the State Bureau of Surveying and Mapping (https://www.tianditu.gov.cn/, accessed on 21 March 2024) was used for the base map analysis [20]. The selected environmental variables were incorporated into the MaxEnt modeling framework to project the potential distribution ranges of plant species under contemporary climatic conditions. To evaluate model accuracy, we implemented a stratified data-partitioning approach in which 75% of the occurrence records were allocated for model training and the remaining 25% served as validation data [21]. All unspecified parameters maintained their default configurations to objectively assess the relative contributions of environmental predictors. Model performance was quantified through receiver operating characteristic (ROC) curve analysis, with the area under the curve (AUC) serving as the primary evaluation metric according to the established methodology [22]. The AUC values, ranging theoretically from 0 (random prediction) to 1 (perfect discrimination), were interpreted using the following categorical scale: ≤0.60 indicated inadequate predictive capacity; 0.61–0.70 represented low accuracy; 0.71–0.80 denoted moderate performance; 0.81–0.90 signified high predictive power; and >0.90 reflected exceptional model precision, as standardized in the ecological modeling literature [23]. This non-parametric threshold-dependent measure demonstrates a positive correlation between AUC magnitude and model reliability [24].

### 2.5. Changes in Spatial Patterns and Center-of-Distribution Analyses in Species Habitat Areas

The suitability areas were binarized in ArcGIS. The areas with a distribution probability < *p* and a value of 0 were classified as unfit, whereas areas with a distribution probability ≥ *p* and a value of 1 were classified as fit. Bivariate maps of unsuitable and suitable areas were generated for each time period [25]. The classifications were as follows: 0–0 indicated unfit areas, 0–1 indicated newly suitable areas, 1–0 indicated loss of suitable areas, and 1–1 indicated retained suitable areas. Changes in the area, trends, and ranges of *Plantago* were calculated under different suitability classes for various climatic scenarios and contemporary conditions [26]. The resulting analyses provided information on areas of expansion, retention, and contraction, as well as shifts in geographic ranges [27].

Building upon these bivariate maps, the SDM toolbox was employed to compute and simulate shifts in the geometric center of potentially suitable habitats across different time periods. This approach facilitated a comparative assessment of the overall directional trends in *Plantago*’s core suitable areas over time, thereby revealing the influence of environmental changes on this plant’s distribution dynamics.

## 3. Results

The MaxEnt model and ROC curve analysis were used to predict the potentially suitable habitats for *Plantago* under current climate conditions and assess the stability and accuracy of the model. The model demonstrated high stability and predictive accuracy, with AUC values exceeding 0.9. The MaxEnt model analysis revealed that environmental factors, particularly precipitation and human activities, had the greatest influence on the distribution of *Plantago*, contributing 58.8% and 50.4%, respectively. The center-of-distribution migration analyses revealed that, due to climate change, the suitable habitats of *Plantago* have expanded toward higher latitudes. Additionally, the distribution of species such as *P. virginica* has also expanded, indicating that climate warming enhanced the success of *P. virginica* as an invasive species.

### 3.1. Accuracy and Evaluation of the Model

An ROC curve analysis was used to validate the prediction results for the potential habitat distribution of *Plantago* under the current climatic conditions (Table 2). The mean AUC values for the training and test sets over 10 repetitions were greater than 0.9. This indicates that model demonstrated high stability and accuracy, making it suitable for predicting the potential distribution of *Plantago.*

### 3.2. Dominant Environmental Factors Affecting Distribution

The MaxEnt model simulation results indicate that the findings from the knife-cut method align with the model’s analysis of the contributions of environmental factors (Table 3). This consistency suggests that the primary environmental factors influencing distribution are precipitation and ha, with shares of 58.8% and 50.4%, respectively. Among the fourteen *Plantago.* species, the dominant environmental factor for four species was human activities (ha) and precipitation for six species. This finding indicates that both precipitation and ha are key factors influencing the distribution of *Plantago*. For example, for *P. aristata*, when precipitation reached 259.51 mm during the warmest quarter, the probability of species occurrence was 50%. As the amount of precipitation increased to 338.67 mm, the occurrence probability increased to 58.8%. However, when the precipitation in the warmest quarter increased further, to 351.87 mm, the occurrence probability decreased to 50%. When precipitation exceeded 358.49 mm, the probability of *P. aristata* occurrence decreased to less than 50%. Therefore, precipitation in the range of 259.51–338.67 mm during the warmest quarter is optimal for the growth of *P. aristata.*

**Figure 1:**
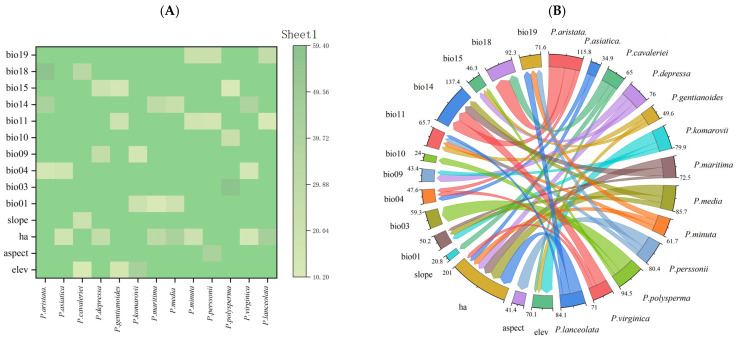
Correlation heat map (**A**) and chord diagram (**B**) of the contribution of environmental factors.

Figure 1 provides a detailed visualization of the environmental variables influencing *Plantago* species distribution according to the MaxEnt model. Figure 1A presents a correlation heat map that clearly illustrates the contribution values of the major environmental variables utilized in the MaxEnt model for each species. Figure 1B, a chord diagram, demonstrates the relative contribution of each environmental variable to the distribution of *Plantago* species. The primary factors are highlighted by the thickest chords, with precipitation in the driest month (bio14) at 58.8% and human activities at 50.4%, underscoring their dominant impact. For secondary factors, elevation and slope have minimal effects on xerophytic species like *P. arachnoidea* (10–20%), which is consistent with their adaptations to arid, high-elevation habitats. Regarding factors specific to invasive species, human activities indicate a strong correlation with *P. lanceolata* (45.7%), emphasizing this factor’s reliance on disturbances from human activities.

### 3.3. Potential Habitat Areas of the Genus Plantago Under Current and Future Climate Scenarios

**Figure 2:**
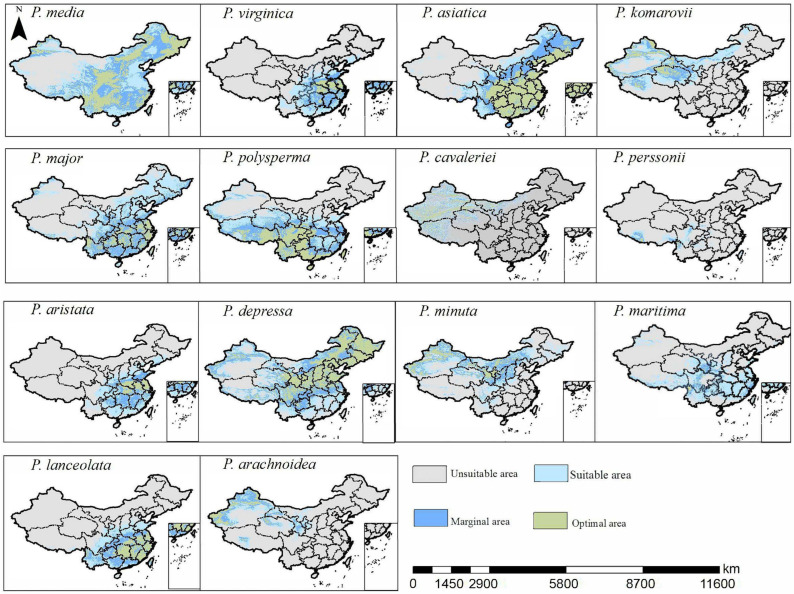
Potential distribution areas of the genus *Plantago* species under the current climate scenario.

The potential suitable habitats for *Plantago* species are distributed nationwide, with northwestern China representing the least suitable region (Figure 2). Notably, certain species, such as *P. arachnoidea,* possess exceptional adaptability to northwestern conditions. *P. depressa* occupies the largest optimal habitat range, covering 30.0% of China’s territory (2.91 million km^2^), predominantly concentrated in the northeastern and central regions, with smaller distributions in northwestern and southeastern areas. In contrast, *P. komarovii* has the smallest optimal suitable habitat (0.556 million km^2^, 5.7% of the national territory), which is primarily localized in southwestern China. Among invasive species, *P. virginica* occupies 1.36 million km^2^ (14.1% of national area), followed by *P. aristata* (1.35 million km^2^, 13.9%) and *P. lanceolata* (1.175 million km^2^, 12.2%). Human activities dominate habitat suitability for *P. major* (2.75 million km^2^, 28.6%), *P. maritima* (2.32 million km^2^, 24.1%), and *P. media* (1.178 million km^2^, 12.27%), demonstrating the significant human influence on these species’ distributions (Table 3 and Table 4).

### 3.4. Changes in the Spatial Distribution of the Genus Under Different Climate Scenarios

Human activities dominate habitat suitability for *P. polysperma*, *P. major*, *P. maritima*, and *P. media* (Table 4). All four species forecast reduced total potential distribution areas compared to current distributions, with the most pronounced declines occurring under the 2090s SSP585 scenario. Quantitatively, *P. polysperma* demonstrates the greatest absolute reduction (167.3 km^2^, −65%), followed by *P. major* (156.4 km^2^, −41%), *P. media* (40.87 km^2^, −5.1%), and *P. maritima* (23.7 km^2^, −4.8%). In contrast, *P. asiatica,* the most widespread *Plantago* species, is projected to maintain stable distribution patterns relative to current conditions. The three invasive species (*P. virginica*, *P. aristata*, and *P. lanceolata*) collectively show a decrease in potential distribution area under the SSP585 scenario, with *P. aristata* experiencing the most substantial proportional decline (−4.6%, 24.9 km^2^) and *P. lanceolata* exhibiting the smallest gradual reduction (−52.4%, 82.5 km^2^). Notably, *P. virginica* displays intermediate vulnerability (−42.5%, 82.6 km^2^). Conversely, *P. komarovii* demonstrates a unique expansion trend under future climate scenarios.

### 3.5. Changes in the Distribution Centroid in the Potential Distribution Area of the Genus Plantago Under Climate Scenarios

Three invasive species exhibited notable centroid displacements over time. *P. aristata* migrated is projected to migrate from 112°56′20″ E, 30°51′56″ N to 113°40′05″ E, 32°19′43″ N; *P. virginica* from 112°40′58″ E, 30°28′26″ N to 113°39′22″ E, 27°15′20″ N; and *P. lanceolata* is projected to move from 110°49′08″ E, 29°03′19″ N to 110°12′13″ E, 29°34′28″ N. These movements collectively demonstrate a poleward migration trend. In contrast, widely distributed species (*P. asiatica*, *P. major*, and *P. depressa*) were found to maintain stable habitat ranges, while northwestern/southwestern/northeastern species (*P. arachnoidea* and *P. minuta*) experience eastward expansions in range. A temporal analysis of migration patterns under different climate projections indicates greater displacement magnitudes during the 2030s under the SSP245 scenario compared to the reduced migration distances projected for the 2090s under the SSP585 scenario, suggesting potential nonlinear responses to varying climate-forcing intensities.

**Figure 3:**
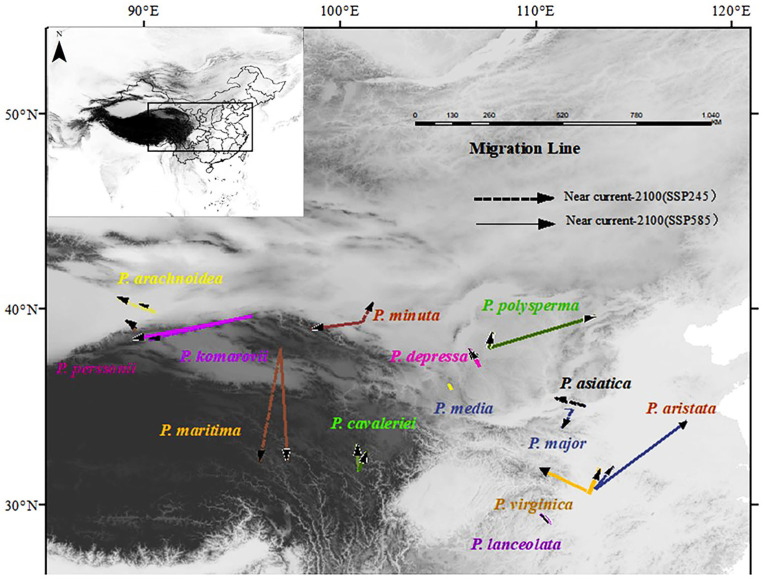
Mass center migration routes of 14 species under SSP245 and SSP585.

*P. aristata* exhibits the longest displacement from its initial coordinates (112°59′48″ E, 30°50′01″ N) to (117°39′36″ E, 34°19′12″ N). Conversely, *P. perssonii* has the shortest range shift, from (89°32′56″ E, 39°01′24″ N) to (89°25′39″ E, 38°58′40″ N). *P. virginica*—a representative invasive species—demonstrates notable reproductive vigor, as evidenced by its progressive range expansion under warming climatic conditions (Figure 3).

### 3.6. Niche Comparisons

**Figure 4:**
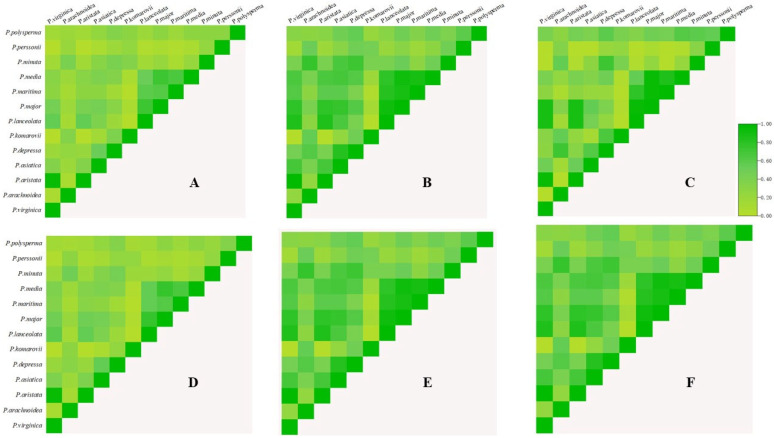
Ecological niche overlap and range overlap among *Plantago* species in the recent past. (**A**) *D* of *Plantago* species; (**B**) *I* of *Plantago* species; (**C**) range overlap of *Plantago* species. Ecological niche overlap and range overlap among *Plantago* species in 2080; (**D**) *D* of *Plantago* species; (**E**) *I* of *Plantago* species; (**F**) range overlap of *Plantago* species.

The niche overlap (*D*) and range overlap (*I*) patterns among the 14 *Plantago* species were analyzed using ENMTools under current and future climate scenarios (Figure 4). Current conditions revealed a particularly high ecological overlap between *P. aristata* and *P. virginica* (*D* = 0.96, *I* = 0.99), while *P. major* demonstrated substantial range overlaps with *P. lanceolata* (*I* = 0.92) and *P. media* (*I* = 0.92). Significant contemporary overlaps included *P. lanceolata*–*P. virginica* (*I* = 0.883), *P. lanceolata*–*P. aristata* (*I* = 0.887), *P. major*-*P. aristata* (*I* = 0.89), *P. major*–*P. lanceolata* (*I* = 0.93), *P. maritima*–*P. major* (*I* = 0.96), and *P. major*–*P. media* (*I* = 0.95). Projections to 2080 showed nuanced changes with slight decreases in *P. aristata*–*P.virginica* overlaps (*D* = 0.94, *I* = 0.99) contrasted by the increased *P. major*–*P. lanceolata* overlap (*I* = 0.93). Emerging future overlaps included *P. major*–*P. komarovii* (*I* = 0.96), *P. major*–*P. lanceolata* (*I* = 0.92), *P. depressa*–*P. media* (*I* = 0.84), and an undocumented *P. media*–*P. virginica* interaction (*I* = 0.95), suggesting the dynamic reorganization of species associations under climate change.

## 4. Discussion

The impact of climate change on the ecological niches of existing plants, particularly in relation to the invasion of *Plantago* species, remains a subject of debate [27,28]. Utilizing three decades of phenological observations from five typical alpine herbaceous species, including *P. arachnidea*, this study found that, despite pronounced climate change, the greening dates of these species exhibited minimal changes. In contrast, the yellowing dates of four species were significantly delayed [29,30]. These results suggest that climate change may modify the phenological patterns of alpine herbaceous plants, such as *P. asiatica*, potentially disrupting their ecological niches and the structure and functionality of alpine grassland plant communities [31].

Emerging studies on climate change impacts concerning invasive flora, exemplified by *P. virginica*, indicate that global change drivers such as thermal elevation and atmospheric nitrogen deposition may significantly modulate biological invasion patterns [32]. While these environmental stressors demonstrate measurable influence on the establishment of invasive species, critical knowledge gaps persist regarding their species-specific impacts on plant performance metrics and interspecific dynamics with indigenous competitors. A controlled phytotron investigation comparing native and invasive *P. asiatica* ecotypes demonstrated that thermal amplification substantially impaired the invasive populations’ competitive indices. This empirical evidence suggests heightened thermal sensitivity in invasive phenotypes compared to autochthonous vegetation [33]. This potentially attenuates the ecological competitiveness of such species against *P. asiatica*, consequently ameliorating the encroachment pressures on native phytocoenoses [34].

Under sustained thermal escalation, *P. asiatica* exhibits pronounced range retraction accompanied by niche compression. These biogeographical patterns suggest that climate modifications exert dual effects. Firstly, they exacerbate *P. asiatica*’s thermal vulnerability while concurrently restructuring the distribution matrices and competitive performance of invasive congeners, including *P. virginica*, *P. aristata*, and *P. lanceolata*. Paradoxically, elevated temperatures diminish invasive species’ competitive efficacy, and simultaneously constrain *P. asiatica*’s biogeographical dominance. This factor highlights the compound climatic threats to this species’ fundamental niche stability through both direct thermal stress and indirect interspecific competition dynamics.

The impact of the future climate on Chinese *Plantago* species shows considerable variability. Studies have demonstrated that environmental changes induced by climate warming are significant drivers influencing the spatial distribution patterns of plants. The underlying mechanisms are closely linked to temperature conditions and precipitation characteristics during key stages of plant growth [35]. In this study, the MaxEnt model was used to generate potential distribution maps for 14 *Plantago* species, identifying the key environmental factors driving distribution changes within the genus. The results indicate that precipitation during the driest month (bio14) and human activities are the most significant factors influencing these distribution shifts. The three invasive *Plantago* species—*P. aristata*, *P. lanceolata*, and *P. virginica*—respond differently to environmental drivers due to their unique ecological strategies, niche preferences, and invasion mechanisms. *P. aristata* is dominated by bio18, thriving in regions with moderate precipitation during the warmest growing season, while excessive precipitation reduces its suitability. These species exploit disturbed habitats such as roadsides and fallow fields, adapting to the transient wet periods in arid or semi-arid areas. In contrast, *P. lanceolata* is dominated by human activities, tolerating such disturbances well and colonizing fragmented landscapes such as urban areas and agricultural edges. *P. lanceolata*’s success correlates with land use intensity and human-mediated dispersal. *P. virginica* is dominated by bio14, exhibiting drought tolerance and relying on the minimal moisture during the driest month to compete with native flora. This species invades grasslands and open woodlands, where dry periods suppress native competitors. These differences stem from niche partitioning and invasion pathway specialization. *P. aristata* and *P. virginica* have narrower climatic tolerances (bio18 and bio14), while *P. lanceolata* shows broader adaptability to human activities. Human activities favor *P. lanceolata* via dispersal vectors such as contaminated soil, whereas *P. aristata* and *P. virginica* mainly rely on climate-mediated range shifts. *P. virginica’s* drought tolerance reduces competition in arid microsites, while *P. aristata* avoids competition by using short-term wet niches. Zheng et al. [36] studied four species from the Chinese *Curcuma* genus and reported that their distributions are primarily influenced by precipitation and temperature, with precipitation having a greater contribution than temperature. Additionally, the moisture-loving characteristics of these four *Lanceolata* species align with those of the *Plantago* species [37]. On the one hand, these results support the reliability and accuracy of the model in predicting suitable habitats for *Plantago* species. The model’s projections suggest that *Plantago* species, particularly *P. lanceolata* and *P. minor*, will find the remaining habitats suitable for survival in northwestern China in the 2090s.

Human activities induce divergent effects on species distribution patterns. Notably, the illustrated distributional shifts reveal the eastward expansion of widespread species *P. arachnoidea* and narrow endemic *P. minuta*, contrasting with the northward spread of the invasive *P. lanceolata*. This pattern corroborates previous findings showing similar influences of human activities on *P. major* and *P. polysperma* distributions. Concurrently, climate change projections suggest that most species will undergo distributional shifts toward higher latitudes and elevations as global warming intensifies [38]. The contemporary literature consistently identifies human activities as critical determinants of species distributions while underscoring the imperative for biodiversity conservation [39]. The direct modification of plant species’ spatial arrangements and diversity profiles via human intervention necessitates the integration of human activity variables into predictive models to enhance accuracy in forecasting future distribution patterns [40]. Importantly, statistical analyses reveal significant negative correlations between the ha indices and potential distribution ranges of *Plantago* species [41], suggesting that human activity pressures may constrain habitat suitability for this genus.

Li et al. [42] conducted ecological conservation research in the Qilian Mountain region and documented sparse human population density, vegetation coverage exceeding 50%, and limited large-scale anthropogenic development. Road networks on the Sichuan Yunnan Plateau facilitated the dispersal of invasive *Elymus nutans* (1.5× seed efficiency over natives), reducing native *Kobresia humilis* cover by 18% in alpine meadows. Light competition dynamics on the Tibetan Plateau show semi-evergreen *Potentilla anserina* reducing the understory by 60%, excluding shade-intolerant herbs, while deciduous shrubs promoted light-adaptive species such as *Gueldenstaedtia diversifolia* [43]. These conditions indicate the exceptional sensitivity of regional biota to human disturbances, a conclusion corroborated by our findings. Predictive modeling reveals that anthropogenic factors constitute the dominant environmental influence for four *Plantago* species (*P. maritima*, *P. media*, *P. minuta*, and *P. perssonii*) among the fourteen studied. This pattern aligns with the established understanding that climate change, coupled with anthropogenic pressures (including coastal land development and the rising sea-level), acts as the primary driver of vegetation reduction in coastal wetland ecosystems. Compelling evidence confirms that human activities significantly reshape *Plantago* distribution patterns, substantiating their pivotal role in driving macroscale species redistribution through range contraction or expansion [44]. Notably, the relative impacts of human activities on three invasive *Plantago* taxa within this study remain debated. Some research emphasizes socioeconomic factors as critical determinants of exotic species distributions [45], while contrasting evidence suggests climatic variables exert a greater influence than human activities on invasive species’ biogeography [46]. Our analysis demonstrates stronger climatic correlations for these three invasive *Plantago* species, as is quantitatively shown in Table 3. Crucially, all climate scenario projections indicate latitudinal distribution centroid shifts toward higher latitudes across the 14 studied species.

Climate change and human activities have driven shifts in species’ ecological niches toward higher altitudes and polar regions, resulting in significant niche overlap among sympatric species. Current data reveal an exceptionally high ecological niche overlap between *P. aristata* and *P. virginica* (*D* > 0.95), with an association strength approaching the maximum values, indicative of intense interspecific competition. Projections suggest a reduced niche overlap between *P. aristata* and both *P. lanceolata* and *P. virginica* in future scenarios. Nevertheless, the strength of the association between *P. lanceolata* and *P. major* shows a slight intensification (*I* = 0.93), concurrent with a contraction in the breadth of *P. major*’s niche.

The displacement of ecological niches to elevational or polar extremes through climatic and human activities pressures may precipitate niche convergence among phylogenetically related species [47]. Our analysis of three invasive taxa (*P. aristata*, *P. virginica*, and *P. lanceolata*) demonstrates a current hyper-overlap (*D* = 0.95) between *P. aristata* and *P. virginica*, coupled with reduced niche dimensions in *P. major*. The maximal overlap values (*D* > 0.95) and near-peak association strength between *P. aristata* and *P. virginica* suggest severe resource competition stemming from shared habitat requirements. Such niche overlap among ecologically similar species may drive future resource-partitioning strategies to alleviate competition and enable coexistence.

Notably, *P. aristata* and *P. lanceolata* exhibit a moderate–high association strength (0.88) with partial niche overlap, suggesting comparatively weaker competitive pressures than those observed with *P. virginica*. Projected declines in distribution areas (as visualized in the results and figures) correlate with the decreasing overlap values between *P. aristata* and both *P. lanceolata* and *P. virginica*, while the overlap intensifies between *P. lanceolata* and *P. virginica*. The current association strength between *P. lanceolata* and native *P. major* (0.9) is projected to increase to 0.93 under the future scenario. This result coincides with climate-mediated reductions in *P. major*’s niche area, a pattern potentially driven by shifting resource availability, which fosters stronger species interactions [48]. Invasive *Plantago* species pose significant threats to native ecosystems through competitive exclusion and other mechanisms. The high niche overlap between *P. aristata* and *P. virginica* (*D* = 0.95) indicates intense competition for shared resources, such as soil moisture and light. While *P. virginica* has a higher reproductive output than native species such as *P. asiatica*, its competitive ability in established ecosystems is often inferior. *P. asiatica*’s stronger resource acquisition strategies, such as a higher nutrient allocation to leaf biomass, allow it to outcompete *P. virginica* in mixed populations. However, under warming scenarios, *P. virginica*’s thermal tolerance and the rapid colonization of disturbed habitats may give it an advantage, particularly in fragmented landscapes where pressures from human activities degrade native resilience. This phenomenon aligns with global patterns in which where invasive plants exploit climate driven habitat shifts to displace natives [49].

Invasive *Plantago* species can also alter soil microbial communities, affecting nutrient cycling. For example, *P. virginica*’s dense root exudates may suppress arbuscular mycorrhizal fungi (AMF), which are critical for native plant symbiosis. This process reduces soil phosphorus availability and disadvantages AMF-dependent species. Similarly, *P. aristata*’s preference for nitrogen-rich soils may accelerate nutrient-leaching in degraded ecosystems, exacerbating soil impoverishment and favoring further invasion [50].

The expansion of invasive *Plantago* species correlates with declines in native floral diversity. *P. virginica*’s high seed output (up to 15,000 seeds/m^2^) creates monoculture patches that exclude understory herbs, reducing pollinator diversity due to floral resource homogenization [51]. This phenomenon mirrors global trends in which invasive species contribute to 60% of documented extinctions, often through niche monopolization and habitat alteration [52]. Furthermore, invasive *Plantago* may disrupt ecosystem services. For example, *P. lanceolata* reduces forage quality in grasslands, which may impact herbivore populations and pastoral economies [53].

In addition, the low association strength (0.63) between *P. lanceolata* and native *P. komarovii* reflects either niche divergence or diminished competitive pressures under future climates. Crucially, the stable spatial dimensions of *P. komarovii*’s niche (as evidenced by the figure results) suggest that *P. lanceolata* is unlikely to pose significant ecological threats to this native congener in future climate scenarios.

The MaxEnt model offers robust predictions for *Plantago* distributions under climate scenarios and human activities, yet still retains some limitations. Our results suggest that trait–climate interactions play a role. For instance, under the SSP126 scenario, *P. virginica* benefits from moderate warming (bio10) due to rapid germination and a short lifecycle, allowing it to expand into fragmented habitats. Meanwhile, *P. depressa* maintains stable ranges through vegetative reproduction, which is supported by low human activity pressures. Under the SSP585 scenario, *P. arachnoidea* leverages CAM photosynthesis to expand into arid northwest China, outcompeting C3 native species [54]. However, *P. maritima* faces coastal habitat loss from the sea-level rises and is forced to migrate inland. However, this phenomenon is limited through competition with non-halophytic invasives. These patterns are consistent with global observations that R-selected species dominate under rapid environmental change, while K-selected species decline, without adaptive traits. To bridge the gap between correlative models and trait ecology, we propose a framework that includes trait environment covariates, dynamic dispersal kernels, and trait-weighted suitability [55]. However, the model assumes static species–environment relationships and ignores dynamic biotic interactions such as competition, which may lead to overestimations of habitat suitability where biotic pressures constrain establishment. This model also presumes fixed ecological niches and disregards potential evolutionary adaptations or phenotypic plasticity under rapid climate change. Spatial biases in the occurrence data, especially for rare species, may also inflate model uncertainty [56]. Additionally, the human activities index aggregates diverse anthropogenic pressures into a single metric, thereby obscuring sector-specific effects. Lastly, the model assumes unlimited dispersal, neglecting geographic barriers that could impede migration.

## 5. Conclusions

Our study demonstrates that climate change and human activities synergistically drive poleward habitat shifts in species of the genus *Plantago* across China, with distinct outcomes for native and invasive taxa. Rising temperatures and precipitation extremes (bio14 and bio18) are the dominant climatic drivers, forcing 79% of studied species to shift their distribution centroids northward by 2100 under high-emission scenarios (SSP585). Invasive species like *P. virginica* exhibit accelerated range expansions, leveraging climate warming to colonize fragmented landscapes. Anthropogenic pressures amplify habitat suitability for invasive species (e.g., *P. lanceolata*: +35% suitability under SSP585) while fragmenting native refugia. Despite the high niche overlap (e.g., *D* = 0.95 between invasive *P. aristata* and *P. virginica*), invasive species monopolize novel habitats under warming scenarios, while native congeners face range contractions (15–40%) due to asymmetric competitive exclusion. These patterns validate the following: (1) trait-mediated climate adaptation governs migration rates, (2) human activities create niche opportunities favoring invasive species, (3) synergistic climate–human pressures accelerate biodiversity loss beyond critical thresholds (SSP245). For invasive species such as *P. virginica* and *P. lanceolata*, it is essential to develop and implement surveillance plans for prevention and control. The findings of this study offer valuable insights into the formulation of long-term conservation and management strategies for the genus *Plantago*.

## Figures and Tables

**Table 1:** Description of the 23 environmental factors for the species distribution model.

Variable	Description
bio1	Mean annual air temperature
bio2	Monthly mean
bio3	Isothermality
bio4	Variation in temperature seasonlity
bio5	Maximum temperature of warmest month
bio6	Minimum temperature of coldest month
bio7	Temperature annual range
bio8	Mean temperature of wettest quarter
bio9	Mean temperature of driest quarter
bio10	Mean temperature of warmest quarter
bio11	Mean temperature of coldest quarter
bio12	Mean annual precipitation
bio13	Precipitation of wettest month
bio14	Precipitation of the driest month
bio15	Variation in precipitation seasonlity
bio16	Precipitation of wettest quarter
bio17	Precipitation of driest quarter
bio18	Precipitation of warmest quarter
bio19	Precipitation of coldest quarter
elev	Elevation
aspect	Influences microclimatic conditions critical to plant growth
ha	Human activity factor
slope	Slope

**Table 2:** Description of MaxEnt simulation predictions for each species distribution model.

Species	Training AUC	Test AUC
*P. arachnoidea*	0.998	0.991
*P. aristata*	0.920	0.919
*P. asiatica*	0.879	0.875
*P. cavaleriei*	0.988	0.990
*P. depressa*	0.912	0.917
*P. komarovii*	0.992	0.997
*P. lanceolata*	0.846	0.847
*P. major*	0.829	0.828
*P. maritima*	0.909	0.911
*P.media*	0.843	0.842
*P. minuta*	0.983	0.976
*P. perssonii*	0.999	0.978
*P. polysperma*	0.972	0.942
*P. virginica*	0.920	0.923

**Table 3:** Description of the results of the analysis of the contribution of different environmental factors to different species.

Species	elev	aspect	ha	slope	bio01	bio03	bio04	bio09	bio10	bio11	bio14	bio15	bio18	bio19	Suitability More Than 50%
*P. arachnoidea*	**38.8**	—	—	20.8	—	—	10.8	—	—	—	—	—	—	—	690–3520 m
*P. aristata.*	—	—	—	—	—	—	15	—	—	—	42	—	**58.8**	—	52.98–97.20 mm
*P. asiatica*	—	—	17.4	—	—	—	**17.5**	—	—	—	—	—	—	—	266.10–279.29 mm
*P. cavaleriei*	10.7	—	—	20.8	—	—	—	—	—	—	—	—	**33.5**	—	5.067–5.26 °C
*P. depressa*	—	—	27.4	—	—	—	—	**27.9**	—	—	—	20.7	—	—	1.00–1.96 °C
*P. komarovii*	**44**	—	—	—	20.4	—	—	15.5	—	—	—	—	—	—	1603.12–1617.25 m
*P. lanceolata*	—	—	**45.7**	—	—	—	—	—	—	11.6	—	—	—	26.8	26.94–27.37 mm
*P. major*	—	—	**50.4**	—	9.3	—	—	—	—	19.8	24.7	14.4	—	—	68.4–72.3
*P. maritima*	—	—	**32**	—	10.3	—	—	—	—	—	30.2	—	—	—	45.49–46.104
*P. media*	—	—	**42**	—	19.5	—	—	—	—	—	24.2	—	—	—	39.372–207.67
*P. minuta*	—	—	21.6	—	—	—	—	—	—	17.7	—	—	—	**22.4**	10.24–17.92 mm
*P. perssonii*	—	**41.4**	—	—	—	—	—	—	—	16.6	—	—	—	22.4	53.4–54.672
*P. polysperma*	—	—	—	—	—	**59.3**	—	—	24	—	—	11.2	—	—	22.67–22.24
*P. virginica*	—	—	14.9	—	—	—	15.1	—	—	—	**41**	—	—	—	51.79–97.20 mm

Note: Bold is the dominant environmental factor.

**Table 4:** The optimal area of the genus *Plantago* under three climate scenarios (10^4^ km^2^).

Species	Current	2030S			2050S			2070S			2090S		
		SSP126	SSP245	SSP585	SSP126	SSP245	SSP585	SSP126	SSP245	SSP585	SSP126	SSP245	SSP585
*P.arachnoida*	66.1	66.1	86.2	72.5	91.2	77.2	95.1	95.9	98.7	86.1	95.8	98.3	173.2
*P. aristata*	135.1	119.1	135.7	137.6	116.5	132.1	147.0	126.3	137.9	179.2	131.4	164.4	145.2
*P. asiatica*	257.7	166.8	247.8	219.9	219.1	267.2	218.2	249.9	246.2	278.0	242.5	221.3	193.8
*P. cavaleriei*	275.9	266.8	223.0	209.5	216.8	231.3	224.2	159.0	160.8	153.0	162.5	140.2	133.7
*P. depressa*	291.1	240.8	275.0	276.1	248.5	270.5	223.6	220.8	242.7	276.7	266.7	250.1	242.9
*P. komarovii*	55.6	12.8	20.7	15.7	25.5	59.5	41.3	44.4	25.5	164.6	149.5	18.5	24.2
*P. lanceolata*	117.5	232.8	142.6	131.9	136.5	141.1	157.8	150.7	158.8	168.8	149.3	164.3	138.3
*P. major*	275.2	296.2	284.9	273.8	280.0	293.9	305.6	277.7	318.4	323.0	323.0	320.2	321.4
*P. maritima*	232.3	145.8	266.1	214.7	187.1	175.1	147.0	214.7	218.1	165.6	236.8	116.7	105.7
*P. media*	117.8	139.9	226.3	209.0	220.9	199.0	199.0	207.4	199.7	199.9	212.2	198.5	118.2
*P. minuta*	159.2	11.5	165.5	205.0	200.5	188.6	188.6	180.7	198.6	191.8	199.2	174.3	159.2
*P. perssonii*	59.0	61.7	22.5	22.2	24.9	58.0	28.0	136.4	130.1	101.4	124.0	119.9	111.5
*P. polysperma*	290.7	292.7	247.8	272.4	227.5	260.6	213.0	109.1	236.7	264.9	209.7	274.4	95.1
*P. virginica*	136.5	117.8	247.8	131.7	107.7	124.4	175.5	127.4	151.1	147.2	150.3	153.9	105.7

## Data Availability

Data can be made available on reasonable request.
